# Text Message Reminders About Free Transportation for Outpatient Substance Use Disorder Treatment: A Pilot Randomized Encouragement Study

**DOI:** 10.1007/s11606-025-09508-4

**Published:** 2025-04-29

**Authors:** Krisda H. Chaiyachati, Nandita Mitra, David Grande, Sheila Kelley, Kaitlyn Shultz, Rebecca E. Stewart

**Affiliations:** 1 Department of Medicine, The University of Pennsylvania Perelman School of Medicine, 3400 Civic Center Boulevard, Philadelphia, PA 19104 USA; 2 The Leonard Davis Institute of Health Economics, The University of Pennsylvania, Philadelphia, USA; 3 Department of Biostatistics, Epidemiology, and Biostatistics, The University of Pennsylvania, Philadelphia, USA; 4 Department of Pediatrics, Mass General for Children, Boston, MA USA; 5 Department of Psychiatry, The University of Pennsylvania Perelman School of Medicine, Philadelphia, USA

**Keywords:** transportation, social determinants of health, retention in care, appointment completion, substance use disorder

## Abstract

**Introduction:**

Substance use disorders (SUDs) are a major epidemic in the USA. Patients who lack transportation may have limited access to outpatient SUD treatment programs and are less likely to remain in care. Encouraging the use of free, reliable transportation during enrollment is an untested mechanism for greater retention.

**Methods:**

A pragmatic pilot study was conducted in Southwest Ohio between November 2020 and July 2022, among people with SUDs like opioid and alcohol use disorders who enrolled in an outpatient treatment program and screened positive for transportation barriers. Eligible patients were offered free, unlimited transportation for the first 12 weeks of treatment and randomized 1:1 to receive weekly text message reminders to schedule their ride (intervention) versus none (control). The primary outcome was retention in care at 12 weeks. Secondary outcomes included retention at 24 weeks and retention at weeks 12 and 24 when stratifying by those dispensed SUD medications (e.g., buprenorphine/naloxone, buprenorphine, methadone) at the treatment site versus not, and SUD type.

**Results:**

The intervention arm included 171 patients and 190 patients were in the control arm. For retention in care at 12 weeks, there was not a statistically significant difference between intervention (77/171 = 45.0%) and control (81/190 = 42.6%) arms (odds ratio (OR) 1.10; 95% confidence interval (CI) 0.57 to 2.00, *p*=0.69). At 24 weeks, retention was lower for both the intervention (44/171 = 25.7%) and control (54/190 = 28.4%) arms but not statistically significantly different (*p*=0.54). Greater retention at 12 weeks was observed for the intervention versus the control arm among patients dispensed SUD medications but at week 24 there was no statistically significant difference. No differences were observed by SUD types at any time point.

**Conclusion:**

Weekly reminders did not affect overall care retention but did at week 12 among patients dispensed SUD medications.

**Supplementary Information:**

The online version contains supplementary material available at 10.1007/s11606-025-09508-4.

## BACKGROUND

Substance use disorders (SUDs) related to opioid and alcohol use result in substantial disruptions to people’s personal lives and contribute to an alarming number of excessive, preventable deaths in the USA. Ohio has the nation’s fourth highest death rate from SUDs related to opioid use, estimated at 47.2 deaths per 100,000 in 2020,^1^ and Southwest Ohio has a high concentration of SUDs.^2^ For alcohol-related disorders, over 6700 people in Ohio die from excessive alcohol use per year.^3^

Access to outpatient SUD treatment programs is critical, and people face significant barriers even after enrolling. Outpatient treatment programs support SUD patients living at home while receiving medication-based treatment, behavioral therapy, and social supports. Barriers include the lack of geographic proximity and the costs of treatment: out-of-pocket treatment costs, time away from work, and the travel costs of going to and from these appointments.^4,5^ While many outpatient treatment programs have incorporated telemedicine since the COVID- 19 pandemic,^6^ in-person appointments remain the predominant SUD treatment appointment type in the USA.^7^

The lack of transportation — availability of rides and affordability — is a significant health-related social need limiting the accessibility of outpatient SUD treatment programs.^8–10^ Awareness about the availability of non-emergency medical transportation (NEMT) is low among eligible patients, with and without SUDs, estimated at 29% among those who are eligible.^11,12^ Few eligible patients use NEMT. For example, 4.5% of Medicaid beneficiaries in Ohio used NEMT in 2021.^11^ Providing and encouraging NEMT is a potential mechanism for improving retention in outpatient SUD treatment programs among patients who lack transportation. Treatment retention is an important path towards remission.

We conducted a pragmatic pilot study using a randomized encouragement study design^13^ in Southwest Ohio to assess the impact of weekly reminders for free, unlimited rideshare-based transportation service for patients in an SUD treatment program on treatment retention.

## METHODS

### Study Design

This study leveraged an encouragement study design and randomized patients 1:1 using a random number generator to receive either (a) a single notification (“control arm”) at the time of enrollment regarding the availability of free, rideshare-based transportation for any appointment during the first 24 weeks of an outpatient treatment program, or (b) the same initial notification as the control arm plus subsequent weekly text message reminders (“treatment arm”) for 12 weeks after enrollment, encouraging patients to use the transportation service. Eligible patients were randomized between November 9, 2020, and December 28, 2021. The last ride occurred on July 11, 2022.

An encouragement study design was utilized because a study arm which did not offer any transportation services was considered unethical by the treatment centers and the service was historically underused. In an encouragement study design, the association between the encouragement (weekly text message reminders) and the outcome of interest (retention in care) is through an indirect effect (rideshare uptake). BrightView Health managed the outpatient treatment centers, including services such as direct dispensation of SUD medications (buprenorphine/naloxone, buprenorphine, methadone, or a combination), individual and group counseling, peer recovery services, case management, and social services for patients with SUD. If an SUD medication was not dispensed at Brightview, they could have been prescribed at a nearby pharmacy; this was consistently true for alcohol use disorder patients prescribed naltrexone or acamprosate.

The University of Pennsylvania Institutional Review Board determined the protocol to be exempt and granted a waiver of informed consent because the study posed minimal risk to patients.

### Patient Cohort

Eligible patients were adult patients (18 years or older) who screened positive as transportation insecure at the time of enrollment in a SUD treatment program at one of four Brightview-managed outpatient sites in Southwest Ohio. Brightview identified patients as transportation insecure if they answered “Yes” to the following question at the time of enrollment: “In the past 12 months, have you had to miss or delay a medical appointment because you did not have a ride?” (see Appendix Figure A.1).

The four sites were chosen because they had some of the highest volumes of patients with SUDs relative to other locations owned and operated by BrightView Health. Patients were not excluded based on previous enrollment in a BrightView Health program (83% of eligible patients). Patients were not included in the study more than once.

Randomization, assigning patients to study arm, and the coordination of rides were administered by Roundtrip, a digital solution that streamlines patient transportation by connecting care coordinators to a national network of on-demand transportation providers, primarily rideshare services.

### Study Procedures

Eligible patients in both study arms were notified about the availability of free transportation services and had their first ride scheduled by the Brightview Patient Access Center during the enrollment intake process. Within a week after enrolling, eligible patients in both study arms received an initial one-time text message detailing how they could schedule subsequent rides by calling a call center to schedule their rides. Individuals in the intervention arm were subsequently provided weekly text message reminders from weeks 2 through 12 after enrollment, encouraging them to use their free rides (see Appendix Figure A.2).

During the study period, a text message interruption to the study procedures occurred between March 12, 2021, and July 21, 2021. The interruption was identified on July 13, 2021, and was deemed technical — an application programming interface (API) disruption occurred between Roundtrip and a third-party text message vendor. During this period, individuals in the intervention arm were not sent subsequent weekly encouragement text messages to use transportation service. SUD treatment and other study procedures were not interrupted during this period, including enrollment into an outpatient treatment program, transportation insecurity screening, scheduling the initial ride, and the initial text message introducing the transportation service to both study arms.

### Outcomes

All outcomes of interest were measured at the patient level. The primary outcome of interest was retention in care at 12 weeks, defined as attending any appointment type 1 week before or after the 12 th-week post-enrollment.

Secondary outcomes included comparing the intervention versus control arms related to (a) retention in care at 24 weeks (attending any appointment type, one week before or after the 24 th-week post-enrollment); (b) retention in care at 12 weeks and 24 weeks, stratified by those who had their SUD dispensation appointments at a Brightview facility versus not and, separately, by SUD type — opioid use disorder, alcohol, and other (e.g., cannabis, stimulant, and anxiolytics); (c) ride usage volume for the first 12 weeks and weeks 13 to 24, in total and stratified by destination (treatment site, home, or other locations); (d) appointment completion rates for the first 12 weeks and weeks 13 to 24, for any appointment and then by appointment type (medical provider, SUD medication dispensation, therapy, and other appointments).

Appointments included in-person appointments, categorized as medical provider visit, SUD medication dispensation, individual or group counseling, and other appointments (peer recovery services, case management, and social services). Patients were recorded as having attended an appointment so long as it occurred at any Brightview Health site as Brightview Health allows their patients to switch facilities during treatment. Telemedicine appointments, or telephone or video appointments (10.9% of all appointments), were not counted because they were not likely to be impacted by transportation services. However, we conducted a sensitivity analysis by broadening the definition of retention in care to include completed telemedicine appointments when measuring retention at 12 and 24 weeks.

### Statistical Analysis

Given this study’s pragmatic design and pilot intention, 200 patients with SUDs were targeted for enrollment and randomization. This was considered a feasible sample size by Roundtrip and Lyft, who respectively administered and sponsored the rides. When the text message interruption was identified, the enrollment time was extended to allow for the accrual of our planned sample size of 200 who were not impacted by the interruption (i.e., patients not in the first 12 weeks post-enrollment during the interruption period). The planned increased accrual would permit per-protocol sensitivity analyses. The evaluation team was blinded to randomization assignment (i.e., intervention versus control), and the entire evaluation and clinical teams were blinded to outcomes until the primary and secondary outcome analyses were completed.

Patient information, including age, sex, ZIP code, SUD subtype (e.g., opioid use disorder, alcohol use disorder, or other), and appointment data, were obtained from the electronic health record. Race and ethnicity were not extracted because a sizeable proportion of patients’ electronic health records contained missing data, respectively 64% and 65%. ZIP-code level education (percentage with a high school education or lower) and unemployment levels were estimated for each patient by applying 2020 US Census data to the patient’s listed home ZIP code at the time of enrollment.

Standardized mean differences (SMDs) for patient-level demographics and quarter-year of enrollment were used to assess for balance between study arms after randomization. Covariates with an SMD < 0.2 were considered to have a fair balance between arms^14^ and were not included in the regression models as adjusted covariates. To estimate the comparison between study arm assignment and retention in care, ride usage, and appointment completion rates, we conducted logistic regression using robust standard errors and a fixed effect for the initial treatment site. Ride volume usage differences between study arms were estimated using *t*-tests. Primary and secondary analyses were conducted using an intention-to-treat approach, not excluding patients potentially affected by the text message interruption period. Because of the text message interruption period, a per-protocol analysis (i.e., excluding any patient in their first 12 weeks of care during the text message interruption period) was conducted for retention in care at 12 weeks and 24 weeks, and by those who received medication(s) to treat their SUD versus not and, separately, by SUD type.

All tests were two-sided; a *p*-value < 0.05 was considered statistically significantly different. All analyses were conducted using Stata v17 (College Station, TX).

## RESULTS

### Study Sample

The study cohort included 361 patients (Table 1). The mean [standard deviation, SD] age for patients was 40.6 [10.8] years, and 49.7% (171/361) were female. Patients lived in ZIP codes where the mean percent [SD] of the population with a high-school degree or lower was 45.9% [15.0], and unemployment was 7.3% [5.6]. For context, the 2020 national mean for a high-school degree or lower^15^ was 38.2%, and unemployment^15^ was 3.4%. Nearly half (168/361 = 46.5%) of patients had SUD medications dispensed by the treatment site. Most study patients had an opioid-use disorder (257/361 = 71.2%) followed by an alcohol-use disorder (34/361 = 9.4%).

**Table 1:** Baseline Patient Characteristics

Patient characteristics	Overall	Study arm – text message reminder encouragement exposure		Standardized mean difference^e^
		Intervention	Control	
*N*	361	171	190	
Age, mean (SD)	40.6 (10.8)	41.0 (11.0)	40.2 (10.7)	0.07
Sex — female, *n* (%)	178 (49.7)	87 (51.8)	91 (47.9)	0.09
High school degree or lower, mean % of ZIP (SD)^a^	12.8 (8.3)	13.5 (9.1)	12.2 (7.4)	0.16
Unemployed, mean % of ZIP (SD)^a^	7.3 (5.6)	7.8 (5.6)	6.8 (5.6)	0.18
SUD medication(s) dispensed at treatment site, *n* (%)	168 (46.5)	82 (48.0)	86 (45.3)	0.05
Buprenorphine/naloxone	131 (83.9)	69 (84.1)	72 (83.7)	
Buprenorphine	9 (5.4)	3 (3.7)	6 (7.0)	
Multiple medications	6 (3.6)	4 (4.9)	2 (2.3)	
Unknown^b^	12 (7.1)	6 (7.3)	6 (7.0)	
SUD subtype, *n* (%)^c^				
Opioid	257 (71.2)	122 (71.3)	135 (80.8)	0.12
Alcohol	34 (9.4)	17 (9.9)	17 (10.2)	
Other^d^	35 (9.7)	20 (11.7)	15 (9.0)	
Initial appointment quarter-year, *n* (%)				
2020 quarter 4	66 (18.3)	33 (19.3)	33 (17.4)	0.17
2021 quarter 1	73 (20.2)	34 (19.9)	39 (20.5)	
2021 quarter 2	63 (17.5)	26 (15.2)	37 (19.5)	
2021 quarter 3	99 (27.4)	50 (29.2)	49 (25.8)	
2021 quarter 4	59 (16.3)	28 (16.4)	31 (16.3)	
2022 quarter 1	1 (0.3)	0 (0.0)	1 (0.5)	
Assigned treatment site,* n* (%)				
Location 1	49 (13.8)	23 (13.7)	26 (13.9)	0.07
Location 2	62 (17.5)	28 (16.7)	34 (18.2)	
Location 3	68 (19.2)	34 (20.2)	34 (18.2)	
Location 4	136 (38.3)	63 (37.5)	73 (39.0)	
Other	40 (11.3)	20 (11.9)	20 (10.7)	

*SUD*, substance use disorder

^a^Sociodemographics are estimates based on a patient’s ZIP code and US Census data

^b^Twelve patients had been designated as receiving dispensed medications, but the specific SUD medication type was not recorded

^c^SUD subtype was missing for 35 patients in the study cohort (23 in the control arm and 12 in the intervention arm) because they did not attend their first appointment, where a formal assessment of the individual’s treatment needs and substance use disorder diagnosis is made

^d^“Other” SUD subtypes included patients being treated who have addictions or dependency with cannabis, anxiolytics, and stimulants

^e^A standardized mean difference (SMD) of < 0.1 is generally considered to indicate good balance, and an SMD < 0.2 is considered a fair balance of covariates between arms

Post-randomization, the intervention arm consisted of 171 patients, and 190 patients were in the control arm. Study arms were balanced (standardized mean difference < 0.2) across all sociodemographic characteristics, dispensed SUD medications, SUD subtype, the initial appointment’s quarter-year, and initial treatment site.

From weeks 1 to 12, the intervention arm received a mean [SD] of 7.5 [4.2] messages compared to the control arm’s 1.0 [0.6] messages (*p*< 0.001).

### Retention in Care

For the intention-to-treat analysis of the primary outcome (Table 2) of overall retention in care at 12 weeks, the intervention arm did not have a statistically significantly different percentage of those retained (77/171 = 45.0%) as the control arm (81/190 = 42.6%) (odds ratio (OR) 1.10; 95% confidence interval (CI) 0.57 to 2.00, *p*= 0.69). For the secondary analysis of retention in care at 24 weeks, retention was lower for both the intervention (44/171 = 25.7%) and control (54/190 = 28.4%) arms but not statistically significantly different (OR 0.85; 95% CI 0.55 to 1.37, *p*= 0.54). Among patients dispensed SUD medications, retention in care between intervention (63/82 = 76.8%) and control (56/86 = 65.1%) arms was statistically significantly different at week 12 (OR 1.88; 95% CI 1.36 to 2.59, *p*< 0.001) but not at week 24 (OR 0.81; 95% CI 0.45 to 1.45, *p*= 0.47). Retention in care was not statistically significantly different among patients not dispensed SUD medications at weeks 12 or 24. No statistically significant differences were observed for retention in care by SUD subtypes (opioids, alcohol, and other use disorders) at 12 weeks or 24 weeks.

**Table 2:** Retention in Care at Weeks 12 and 24 After Enrollment by Receipt of Medication-Assisted Treatment or Substance Use Disorder Type (Intention-to-Treat)

Categories	Retention in care at week 12		*p*-value	Retention in care at week 24		*p*-value
	Retained *n* (%)	Odds ratio (95% CI)		Retained *n* (%)	Odds ratio (95% CI)	
All patients (*N*= 361)						
Intervention (*n*= 171)	77 (45.0)	1.10 (0.57 to 2.00)	0.69	44 (25.7)	0.86 (0.55 to 1.37)	0.54
Control (*n*= 190)	81 (42.6)			54 (28.4)		
Dispensed SUD medications^a^						
Dispensed (*N*= 168)						
Intervention (*n*= 82)	63 (76.8)	1.88 (1.36 to 2.59)	< 0.001	34 (41.5)	0.81 (0.45 to 1.45)	0.47
Control (*n*= 86)	56 (65.1)			41 (47.7)		
Not dispensed (*N*= 187)						
Intervention (*n*= 89)	14 (15.7)	0.57 (0.24 to 1.35)	0.20	10 (11.2)	0.82 (0.21 to 3.17)	0.77
Control (*n*= 104)	25 (24.0)			13 (12.5)		
SUD type						
Opioid (*N*= 257)						
Intervention (*n*= 122)	66 (54.1)	1.21 (0.64 to 2.29)	0.55	35 (28.7)	0.80 (0.50 to 1.27)	0.35
Control (*n*= 135)	66 (48.9)			45 (33.3)		
Alcohol (*N*= 34)						
Intervention (*n*= 17)	4 (23.5)	0.70 (0.16 to 2.98)	0.62	4 (23.5)	0.90 (0.18 to 4.64)	0.90
Control (*n*= 17)	6 (35.3)			5 (29.4)		
Other^b^ (*N*= 35)						
Intervention (*n*= 20)	7 (35.0)	0.34 (0.10 to 1.12)	0.08	5 (25.0)	0.51 (0.05 to 5.43)	0.57
Control (*n*= 15)	8 (53.3)			3 (20.0)		

*SUD*, substance use disorder

^a^Dispensed SUD medications consisted of buprenorphine/naloxone, buprenorphine, and methadone

^b^“Other” SUD subtypes included patients being treated who have addictions or dependency with cannabis, anxiolytics, and stimulants

The per-protocol analysis (Appendix Table A.1) consisted of a cohort of 218 patients, 105 patients in the intervention arm and 113 in the control arm. Retention in care at 12 weeks was statistically significantly different between the intervention (40/105 = 38.1%) and control arm (35/113 = 31.0%) (OR 1.41; 95% CI 1.07 to 1.86, *p*= 0.01). Among patients dispensed SUD medications, retention in care between intervention (33/46 = 71.4%) and control (24/45 = 53.3%) was statistically significantly different at week 12 (OR 2.26; 95% CI 1.67 to 3.95, *p*< 0.001) but not at week 24 (OR 1.34; 95% CI 0.39 to 4.63, *p*= 0.64). Retention in care for the overall per-protocol cohort at 24 weeks and retention by SUD subtypes at 12 or 24 weeks were not statistically significantly different between study arms. A sensitivity analysis, including completed telemedicine appointments, did not alter our findings for retention in care among the overall per-protocol cohort at weeks 12 and 24.

### Ride Usage Volume

The mean [SD] overall number of rides (Table 3) between weeks 1 and 12 used by the intervention arm was 5.2 [10.8] and 3.9 [5.4] for the control arm (difference, + 1.3 rides; 95% CI − 0.5 to + 3.0, p= 0.15). Between weeks 13 to 24, the number of overall rides for the intervention arm was 2.5 [10.7] and 1.8 [5.4] for the control arm (difference, + 0.7; 95% CI − 1.1 to + 2.4, *p*= 0.45). Stratified by destination — to treatment site, home, or other locations (non-Brightview healthcare facilities, grocery stores, etc.) — ride volumes were not statistically significantly different by treatment arm in the first 12 weeks and weeks 13 to 24. The per-protocol analysis did not result in divergent observations (Appendix Table A.2).

**Table 3:** Ride Volume by Study Arm, Ride Destination, and Time Period (Intention-to-Treat)

	Weeks 1 to 12			Weeks 13 to 24		
	Intervention (*N*= 171) No. (SD)	Control (*N*= 190) No. (SD)	Difference (95% CI) *p*-value	Intervention (*N*= 171) No. (SD)	Control (*N*= 190) No. (SD)	Difference (95% CI) (95% CI)
Ride volume						
Any destination	5.2 (10.8)	3.9 (5.4)	+ 1.3 (− 0.5 to + 3.0) 0.15	2.5 (10.7)	1.8 (5.4)	+ 0.7 (− 1.1 to + 2.4) 0.45
To treatment site	2.3 (2.6)	2.0 (2.8)	+ 0.3 (− 0.3 to + 0.8) 0.35	0.8 (2.3)	0.9 (2.8)	− 0.1 (− 0.6 to + 0.5) 0.82
To home	2.4 (7.0)	1.7 (2.7)	+ 0.7 (− 0.4 to + 1.8) 0.21	1.3 (7.0)	0.8 (2.6)	+ 0.5 (− 0.6 to + 1.6) 0.36
To other locations^a^	0.5 (2.5)	0.2 (0.7)	+ 0.3 (0.0 to + 0.7) 0.08	0.3 (2.4)	0.1 (0.6)	+ 0.2 (− 0.1 to + 0.6) 0.20

^a^“Other locations” included non-Brightview healthcare facilities, grocery stores, pharmacies, courthouses, and churches

### Appointment Completion Rate

The completion rate for any appointment (Table 4) between 1 week and 12 weeks in the intervention arm (40.3%) was not statistically significantly different from the control arm (38.6%) (difference, + 1.7 percentage points, 95% CI − 4.3 to + 7.7, *p*= 0.59). A similar observation occurred for the appointment completion rate comparison between weeks 13 and 24 for any appointment and when stratifying by appointment type (medical provider, SUD medication pickup, therapy, and other services such as peer recovery services and case management) for weeks 1 to 12 and weeks 13 to 14. The per-protocol analysis did not result in divergent observations (Appendix Table A.3).

**Table 4:** Appointment Completion Rates by Study Arm, Appointment Type, and Time Period (Intention-to-Treat)

Study arm by appointment type	No. of study patients with scheduled appointments	Weeks 1 to 12		No. of study patients with scheduled appointments	Weeks 13 to 24	
		Appointment completion rate	Difference (95% CI) *p*-value		Appointment completion rate	Difference (95% CI) *p*-value
Overall						
Intervention	171	40.3	+ 1.7 (− 4.3 to + 7.7)	82	45.5	+ 0.3 (− 8.6 to + 9.1)
Control	189	38.6	0.59	88	45.2	0.95
Medical provider						
Intervention	168	50.0	+ 2.7 (− 4.0 to + 9.4)	70	55.6	+ 1.0 (− 9.6 to + 11.6)
Control	184	47.3	0.42	85	54.6	0.85
Medication pickup						
Intervention	90	92.4	+ 2.4 (− 1.0 to + 5.8)	45	85.2	+ 4.1 (− 4.9 to + 13.1)
Control	98	90.0	0.16	55	81.0	0.36
Therapy^a^						
Intervention	152	36.2	+ 3.1 (− 2.8 to + 8.9)	80	31.5	+ 3.9 (− 13.4 to + 5.7)
Control	168	33.1	0.31	79	35.4	0.42
Other^b^						
Intervention	104	62.7	− 2.2 (− 10.9 to + 6.5)	58	75.9	+ 7.0 (− 4.6 to + 18.6)
Control	109	64.9	0.62	66	68.8	0.23

^a^“Therapy” included both individual and group therapy appointments

^b^“Other” services include peer recovery, case management, and social services

## DISCUSSION

This study has three main findings. First, retention in care was not associated with weekly text messages reminding patients about free transportation in the intention-to-treat analysis. Second, the odds of retention in care at 12 weeks for patients dispensed SUD medications was higher in the intention-to-treat and the per-protocol analysis. Third, we observed no differences in the number of rides used or appointment completion rates between intervention and control patients in the intention-to-treat or per-protocol analyses.

While transportation is a documented barrier to accessing outpatient SUD treatment,^8^ to our knowledge, this is the first published evaluation of a text message reminder to use transportation among patients with SUD. Our results align with a randomized trial of Medicaid beneficiaries in urban West Philadelphia,^16^ finding no association between offering rideshare-based transportation and missed primary care appointments. In-depth qualitative interviews of patients in the trial highlighted how the type of clinical condition being treated influenced their perception of clinical need and desires to attend an appointment or not, independent of their transportation needs.^17^ In contrast to our study, that study did not screen for transportation insecurity.

However, a propensity score-matched study among patients of a Medicare Accountable Care Organization with transportation needs found that NEMT resulted in more outpatient visits, indicating improved access to care in Minneapolis–Saint Paul, Minnesota.^18^ A difference-in-difference analysis of no-show appointment rates observed a significant reduction in no-show rates for clinics whose patients lived close to the new rail line, particularly Medicaid beneficiaries.^19^ Our findings, combined with those from prior studies, highlight how the differences in observed appointment attendance may be due to different transportation needs for specific clinical condition(s), local transportation infrastructure, and insurance types.

This study has limitations. The study may have been inadequately powered to detect a difference. The text message reminders — the primary intervention — were interrupted. Alternative transportation options for patients were not captured and may have impacted our findings. Sociodemographic data, and race and ethnicity data were missing in the electronic health record for a sizeable portion of patients. The study was conducted during the COVID- 19 pandemic when infection control policies and high incidence rates may have resulted in staffing shortages or influenced patients’ care-seeking behavior. However, these center-level and patient-level influences should have been balanced between study arms; yet the generalizability of our findings outside of the pandemic context is limited. These findings may not be generalizable to all SUD treatment programs such as methadone-only treatment programs which have higher retention rates.

Our study likely indicates that, alone, text message reminders encouraging greater utilization of NEMT (free rideshares in our case) will not likely impact overall treatment retention. The content (e.g., non-personalized messages) and quantity of other text messages (e.g., spam messages or additional appointment reminders) could have muted patients’ receptivity to reminders and therefore the impact of our text message intervention. Additional interventions designed to improve the convenience of appointments, such as integrating more telemedicine for therapy to eliminate the time and cost of traveling to appointments, may have greater impacts on care retention than reminders about NEMT. Additionally, estimating the impact of free ridesharing on care retention was untested in our study. Future studies, with a particular focus on patients who have their SUD medications dispensed by treatment sites, may better estimate the impact of free ridesharing on care retention with a control arm not offering rides.

In conclusion, subsequent weekly reminders of free transportation did not influence ride usage and had mixed impacts on overall care retention at 12 weeks and 24. However, retention in care was greater among those dispensed SUD medications from the treatment site.

## Supplementary Information

Below is the link to the electronic supplementary material.

Supplementary file1 (DOCX 32 KB)

## Data Availability

The data that support the findings of this study are available from Roundtrip and Brightview Health, but restrictions apply to the availability of these data, which were used under license for the current study, and so are not publicly available. Data are however available from the authors upon reasonable request and with permission of Roundtrip and Brightview Health.
