# Supplementary material for: Text Message Reminders About Free Transportation for Outpatient Substance Use Disorder Treatment: A Pilot Randomized Encouragement Study
Source: J Gen Intern Med. 2025 Apr 29;41(1):35–42. doi: 10.1007/s11606-025-09508-4 (PMC12855645; doi:10.1007/s11606-025-09508-4)
Supplement: Supplementary file 1 — Supplementary file1 (DOCX 32 KB) [file 11606_2025_9508_MOESM1_ESM.docx]

**Appendix**

**Figure A.1**. Transportation screening questions

1. ​In the past has the lack of transportation kept you from any of the following? (Check all that apply)​

Medical appointments​

Getting medications

​Meetings or appointments (non-medical)

​Work​

Things that I need for my daily life​

I have not had any problems with transportation

​2. If transportation has kept you from any of the above, how often?​

Every time

​Almost every time

​Occasionally​

Almost never

​Never​​​

* NOTE: Only question #1 was used to screen for being offered the ride benefit and, therefore, randomized to either the intervention or control arm.

**Figure A.2.** Text message content

*INITIAL TEXT MESSAGE*: Hi [Rider first name]. Welcome to Brightview! Did you know there is free transportation to appointments? Please call this number to book your ride using Roundtrip for any of your appointments at (513) 647-1933.

*SUBSEQUENT TEXT MESSAGE*: Hi [Rider first name]. Brightview is here to help!  Remember, you have free transportation to appointments. Please call this number to book your ride using Roundtrip for any of your appointments at (513) 647-1933.

*NOTE: The initial text message was sent to both arms of the study—intervention and controls—but the subsequent text messages were sent to the intervention arm only.

**Table A.1.** Retention in care at weeks 12 and 24 after enrollment by receipt of medication assisted treatment or substance-use disorder type (per protocol)

| **Categories** | **Retention in Care**  **at Week 12** | | **P-value** | **Retention in Care**  **at Week 24** | | **P-value** |
| --- | --- | --- | --- | --- | --- | --- |
|  | **Retained**  **n (%)** | **Odds Ratio**  **(95% CI)** |  | **Retained**  **n (%)** | **Odds Ratio**  **(95% CI)** |  |
| **All Patients (N=218)** |  |  |  |  |  |  |
| Intervention (n=105) | 40 (38.1) | 1.41 (1.07 to 1.86) | 0.01 | 26 (24.8) | 1.23 (0.50 to 3.02) | 0.65 |
| Control (n=113) | 35 (31.0) |  |  | 24 (21.2) |  |  |
|  |  |  |  |  |  |  |
| **Dispensed SUD Medications**^a^ |  |  |  |  |  |  |
| **Dispensed (N=91)** |  |  |  |  |  |  |
| Intervention (n=46) | 33 (71.4) | 2.26 (1.67 to 3.05) | <0.001 | 21 (45.7) | 1.34 (0.39 to 4.63) | 0.64 |
| Control (n=45) | 24 (53.3) |  |  | 18 (40.0) |  |  |
|  |  |  |  |  |  |  |
| **Not Dispensed (N=127)** |  |  |  |  |  |  |
| Intervention (n=59) | 7 (11.9) | 0.76 (0.30 to 1.93) | 0.56 | 5 (8.5) | 0.93 (0.18 to 4.92) | 0.93 |
| Control (n=68) | 11 (16.2) |  |  | 6 (8.8) |  |  |
|  |  |  |  |  |  |  |
| **SUD Type** |  |  |  |  |  |  |
| **OUD (N=152)** |  |  |  |  |  |  |
| Intervention (n=73) | 33 (45.2) | 1.43 (0.92 to 2.20) | 0.11 | 21 (28.8) | 1.10 (0.50 to 2.46) | 0.81 |
| Control (n=79) | 29 (36.7) |  |  | 21 (26.6) |  |  |
|  |  |  |  |  |  |  |
| **Alcohol (N=19)** |  |  |  |  |  |  |
| Intervention (n=10) | 1 (10.0) | 1.73 (0.08 to 38.12) | 0.72 | 1 (10.0) | 1.73 (0.08 to 38.12) | 0.72 |
| Control (n=9) | 1 (11.1) |  |  | 1 (11.1) |  |  |
|  |  |  |  |  |  |  |
| **Other^b^ (N=21)** |  |  |  |  |  |  |
| Intervention (n=13) | 6 (46.2) | 0.83 (0.15 to 4.54) | 0.83 | 4 (30.8) | 1.26 (0.02 to 100.54) | 0.92 |
| Control (n=8) | 4 (50.0) |  |  | 1 (12.5) |  |  |

SUD: Substance-use disorder

^a^ Dispensed SUD medications consisted of buprenorphine/naloxone, buprenorphine, and methadone.

^b^ “Other” conditions include patients being treated who have addictions or dependency with cannabis, anxiolytics, and stimulants.

**Table A.2.** Ride volume by study arm, ride destination, and time period (per protocol)

|  | **Week 1 to 12** | | | **Week 13 to 24** | | |
| --- | --- | --- | --- | --- | --- | --- |
|  | Intervention  (N=105)  No. (SD) | Control  (N=113)  No. (SD) | Difference (95% CI)  P-value | Intervention  (N=105)  No. (SD) | Control  (N=113)  No. (SD) | Difference (95% CI)  (95% CI) |
| **Ride volume** |  |  |  |  |  |  |
| Any destination | 5.3 (13.0) | 3.5 (5.5) | +1.8 (-0.8 to +4.5)  0.17 | 2.8 (13.1) | 1.9 (5.6) | +1.0 (-1.7 to +3.6)  0.48 |
| To treatment site | 2.2 (2.6) | 1.8 (2.9) | +0.4 (-0.3 to +1.1)  0.29 | 0.9 (2.3) | 0.9 (2.9) | -0.1 (-0.8 to +0.6)  0.80 |
| To home | 2.5 (8.7) | 1.4 (2.6) | +1.1 (-0.6 to +2.8)  0.20 | 1.6 (8.7) | 0.8 (2.6) | +0.8 (-0.9 to +2.5)  0.37 |
| To other location^a^ | 0.5 (3.1) | 0.2 (0.8) | +0.3 (-0.3 to +0.9)  0.26 | 0.4 (3.1) | 0.1 (0.7) | +0.3 (-0.3 to +0.9)  0.35 |

^a^ “Other” locations included non-Brightview healthcare facilities, grocery stores, pharmacies, courthouses, and churches.

**Table A.3.** Appointment completion rates by study arm, appointment type, and time period (per protocol)

| **Study Arm**  **by Appointment Type** |  | **Week 1 to 12** | |  | **Week 13 to 24** | |
| --- | --- | --- | --- | --- | --- | --- |
|  | **# of Study Patients with Scheduled Appointments** | **Appointment Completion**  **Rate** | **Difference (95% CI)**  **P-value** | **# of Study Patients with Scheduled Appointments** | **Appointment Completion**  **Rate** | **Difference (95% CI)**  **P-value** |
| **Overall** |  |  |  |  |  |  |
| Intervention | 105 | 39.3 | +2.9 (-5.1 to +10.9) | 44 | 45.6 | -11 (-14.4 to +12.2) |
| Control | 112 | 36.5 | 0.47 | 44 | 46.8 | 0.87 |
|  |  |  |  |  |  |  |
| **Medical provider** |  |  |  |  |  |  |
| Intervention | 102 | 49.5 | +4.1 (-3.9 to +13.1) | 38 | 55.7 | +4.2 (-11.3 to +19.7) |
| Control | 107 | 45.4 | 0.37 | 41 | 51.4 | 0.59 |
|  |  |  |  |  |  |  |
| **Medication pickup** |  |  |  |  |  |  |
| Intervention | 51 | 93.2 | +3.7 (-0.9 to +8.3) | 24 | 81.9 | +0.9 (-13.9 to +12.1) |
| Control | 52 | 89.5 | 0.11 | 25 | 82.9 | 0.88 |
|  |  |  |  |  |  |  |
| **Therapy**^a^ |  |  |  |  |  |  |
| Intervention | 94 | 35.2 | +4.2 (-3.6 to +12.0) | 42 | 32.4 | -8.4 (-23.1 to +6.4) |
| Control | 99 | 31.0 | 0.29 | 37 | 40.7 | 0.26 |
|  |  |  |  |  |  |  |
| **Other**^b^ |  |  |  |  |  |  |
| Intervention | 57 | 61.7 | -0.6 (-13.9 to +12.8) | 32 | 78.9 | +7.6 (-8.6 to +23.7) |
| Control | 51 | 62.3 | 0.93 | 30 | 71.4 | 0.35 |

^a^ “Therapy” included both individual and group therapy appointments.

^b^ “Other” services include peer recovery, case management, and social services.
